# Supplementary material for: Genome-wide association studies reveal novel loci associated with pyrethroid and organophosphate resistance in Anopheles gambiae and Anopheles coluzzii
Source: Nat Commun. 2023 Aug 16;14:4946. doi: 10.1038/s41467-023-40693-0 (PMC10432508; doi:10.1038/s41467-023-40693-0)
Supplement: Supplementary file 1 — Supplementary Information [file 41467_2023_40693_MOESM1_ESM.pdf]

Genome-wide association studies reveal novel loci associated with pyrethroid and organophosphate resistance in *Anopheles gambiae s.l.*

Eric R. Lucas, Sanjay C. Nagi, Alexander Egyir-Yawson, John Essandoh, Sam Dadzie, Joseph Chabi, Luc S. Djogbénou, Adandé A. Medjigbodo, Constant V. Edi, Guillaume K. Ketoh, Benjamin G. Koudou, Arjen E. Van't Hof, Emily J. Rippon, Dimitra Pipini, Nicholas J. Harding, Naomi A. Dyer, Louise T. Cerdeira, Chris S. Clarkson, Dominic P. Kwiatkowski, Alistair Miles, Martin J. Donnelly, David Weetman

Electronic Supplementary Material

Supplementary figures and tables

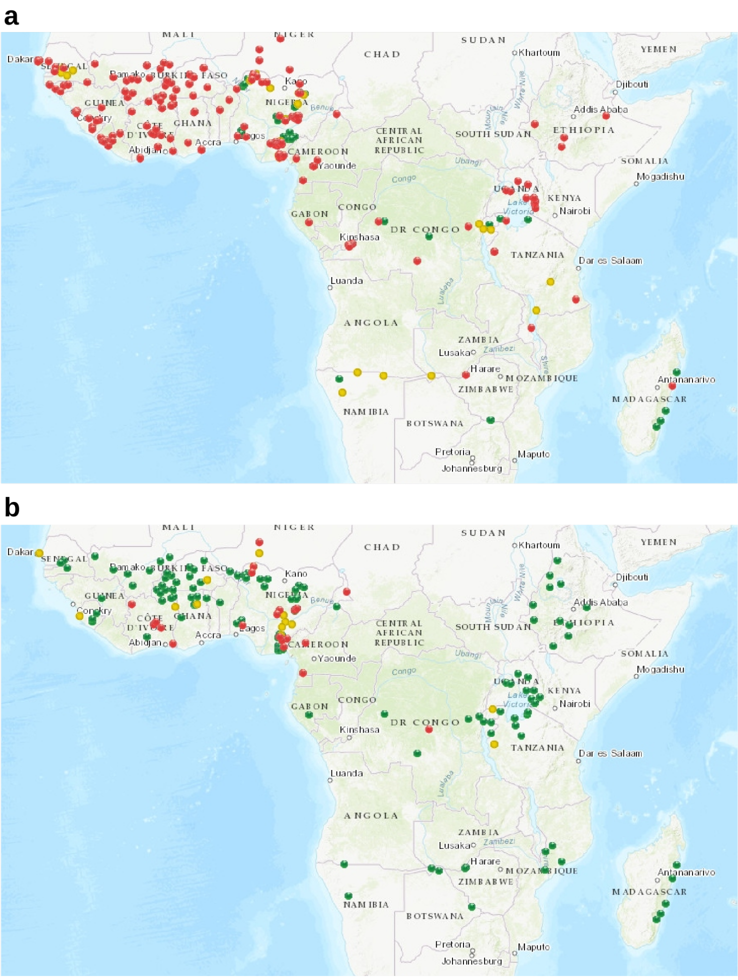

Fig. S1: Distribution of insecticide resistance detected by studies in *An. gambiae* sl over the last 5 years (2017-2022) to deltamethrin (a) and PM (b), indicating confirmed resistance (red), possible resistance (yellow) and susceptibility (green). Data obtained from IR-Mapper v2.0 `www.irmapper.com/` (25th of November 2022)

	Ace1_Dup0	Ace1_Dup1	Ace1_Dup2	Ace1_Del1	Ace1_Del2	Ace1_Del3	Ace1_Del4
Avrankou coluzzii	.05	.05	0	.01	0	0	0
Korle-Bu coluzzii	.85	.87	0	.44	0	0	.06
Aboisso gambiae	.95	.95	0	.95	0	0	0
Baguida gambiae	.99	1	0	.95	0	0	0
Madina gambiae	.9	.92	0	.77	0	0	0
Obuasi gambiae	1	1	0	.98	0	0	0

	Cyp6aap_Dup0	Cyp6aap_Dup1	Cyp6aap_Dup1a	Cyp6aap_Dup1b	Cyp6aap_Dup2	Cyp6aap_Dup3	Cyp6aap_Dup4	Cyp6aap_Dup5	Cyp6aap_Dup6	Cyp6aap_Dup7	Cyp6aap_Dup8	Cyp6aap_Dup9	Cyp6aap_Dup10	Cyp6aap_Dup11	Cyp6aap_Dup12	Cyp6aap_Dup13	Cyp6aap_Dup14	Cyp6aap_Dup15	Cyp6aap_Dup16	Cyp6aap_Dup17	Cyp6aap_Dup18	Cyp6aap_Dup19	Cyp6aap_Dup20	Cyp6aap_Dup21	Cyp6aap_Dup22	Cyp6aap_Dup23	Cyp6aap_Dup24	Cyp6aap_Dup25	Cyp6aap_Dup26	Cyp6aap_Dup27	Cyp6aap_Dup28	Cyp6aap_Dup29	Cyp6aap_Dup30
Avrankou coluzzii	.77	0	0	.01	0	0	0	0	0	.1	0	0	.43	0	0	0	0	0	0	.01	0	0	0	0	0	0	0	0	0	0	0	0	0
Korle-Bu coluzzii	.3	0	0	0	0	0	0	0	0	.004	0	0	.06	.004	0	0	0	0	0	.02	.16	0	0	0	0	0	0	0	0	0	0	0	0
Aboisso gambiae	.11	0	0	0	0	0	0	0	0	0	.03	0	0	0	0	0	0	0	0	0	0	.03	.03	0	0	0	0	0	0	0	0	0	0
Baguida gambiae	.03	0	0	0	0	0	0	0	0	0	0	0	0	0	0	0	0	0	0	0	0	0	0	0	0	0	0	0	0	0	0	0	0
Madina gambiae	.34	0	0	0	0	0	0	0	0	0	.02	0	0	0	0	0	0	0	0	0	0	0	0	0	0	0	0	0	0	0	0	0	0
Obuasi gambiae	.1	0	0	0	0	0	0	0	0	0	.005	0	0	0	0	0	0	0	0	0	0	0	.02	0	0	0	0	0	0	0	0	0	0

	Cyp6mz_Dup0	Cyp6mz_Dupm1	Cyp6mz_Dupm2	Cyp6mz_Dupm3	Cyp6mz_Dupm4	Cyp6mz_Dupm5	Cyp6mz_Dupz1	Cyp6mz_Dupz2	Cyp6mz_Dupz3	Cyp6mz_Dupz4	Cyp6mz_Dupz5	Cyp6mz_Dupmz1
Avrankou coluzzii	.04	0	0	0	0	0	0	0	0	0	0	0
Korle-Bu coluzzii	.06	0	0	0	0	0	0	0	0	0	0	0
Aboisso gambiae	.05	0	0	0	0	0	.03	0	0	0	0	0
Baguida gambiae	.05	0	0	0	0	0	0	0	0	0	0	0
Madina gambiae	.24	0	.006	0	0	0	.11	0	.02	0	0	0
Obuasi gambiae	.2	0	.06	0	0	0	.09	0	0	0	0	0

	Gstue_Dup0	Gstue_Dup1	Gstue_Dup2	Gstue_Dup3	Gstue_Dup4	Gstue_Dup5	Gstue_Dup6	Gstue_Dup7	Gstue_Dup8	Gstue_Dup9	Gstue_Dup10	Gstue_Dup11	Gstue_Dup12	Gstue_Dup13	Gstue_Dup14	Gstue_Dup15	Gstue_Dup16
Avrankou coluzzii	.68	.05	0	0	0	0	0	.52	0	0	0	0	0	0	0	0	0
Korle-Bu coluzzii	.23	0	0	0	0	0	0	.22	0	0	0	0	0	0	0	0	0
Aboisso gambiae	0	0	0	0	0	0	0	0	0	0	0	0	0	0	0	0	0
Baguida gambiae	.01	0	0	0	0	0	0	0	0	0	0	0	0	0	0	0	0
Madina gambiae	.07	0	0	0	0	0	0	0	0	0	0	0	0	0	0	0	0
Obuasi gambiae	.08	0	0	0	0	0	0	0	0	0	0	0	0	0	0	0	0

	Cyp9k1_Dup0	Cyp9k1_Dup1	Cyp9k1_Dup2	Cyp9k1_Dup3	Cyp9k1_Dup4	Cyp9k1_Dup5	Cyp9k1_Dup6	Cyp9k1_Dup7	Cyp9k1_Dup8	Cyp9k1_Dup9	Cyp9k1_Dup10	Cyp9k1_Dup11	Cyp9k1_Dup12	Cyp9k1_Dup13	Cyp9k1_Dup14	Cyp9k1_Dup15	Cyp9k1_Dup16	Cyp9k1_Dup17	Cyp9k1_Dup18	Cyp9k1_Dup19	Cyp9k1_Dup20	Cyp9k1_Dup21	Cyp9k1_Dup22	Cyp9k1_Dup23	Cyp9k1_Dup24	Cyp9k1_Dup25	Cyp9k1_Dup26	Cyp9k1_Dup27	Cyp9k1_Dup28
Avrankou coluzzii	.03	0	0	0	0	0	0	0	0	0	.03	0	0	0	0	0	0	0	0	0	0	0	0	0	0	0	0	0	0
Korle-Bu coluzzii	.16	0	0	0	0	0	0	0	0	0	.17	0	0	0	0	0	0	0	0	0	0	0	0	0	0	0	0	0	0
Aboisso gambiae	.84	0	0	0	0	0	0	0	0	0	0	.16	0	0	0	0	0	0	0	0	0	0	0	0	0	0	0	0	.73
Baguida gambiae	.96	0	0	0	.16	0	0	.16	0	0	.73	.01	0	0	0	0	0	0	0	0	0	0	.02	0	0	0	0	0	.19
Madina gambiae	.93	0	0	0	.1	0	0	.006	0	0	.006	.13	0	.006	0	0	0	0	0	0	0	0	.006	0	0	0	0	0	.86
Obuasi gambiae	.93	0	0	0	.02	0	0	.02	0	0	0	.02	0	0	0	0	0	0	0	0	0	0	.01	0	0	0	0	0	.91

Fig. S2: Frequency (proportion of samples carrying at least one copy) of known CNV alleles detected using diagnostic reads around *Ace1*, the *Cyp6aa / Cyp6p* cluster, the *Cyp6m / Cyp6z* cluster, the *Gste* cluster and *Cyp9k1*. Darkness of blue (*An. gambiae*) and red (*An. coluzzii*) provided as a visual aid for the magnitude of the value in each cell. The genomic coordinates of each CNV allele can be found in Supplementary Data S3 In each cluster, the "Dup0" column indicates the presence of increased copy number in any of the genes in the cluster. Where this is larger than the sum of known alleles, it suggests the presence of CNV alleles not present in the Ag1000G database. "Del" alleles in Ace1 represent secondary deletions within the Ace1-Dup1 CNV.

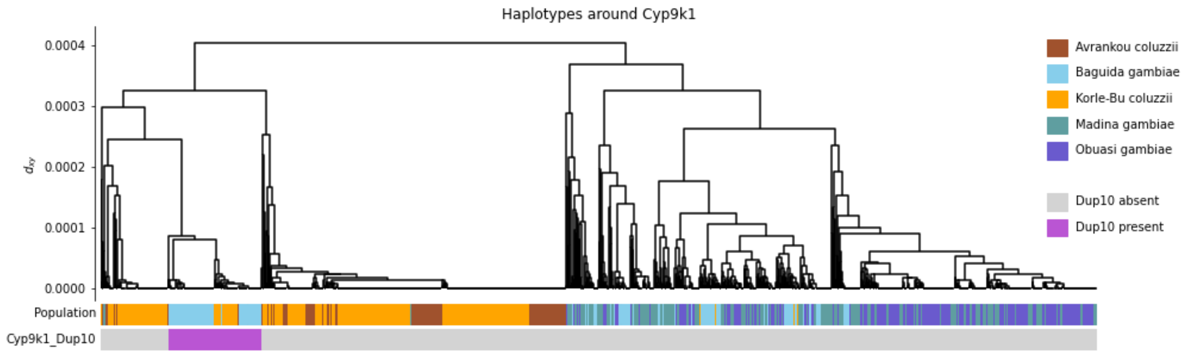

Fig. S3: Haplotypes bearing the CNV allele Cyp9k1_Dup10 in *An. gambiae* and *An. coluzzii* form a single cluster nested within other *An. coluzzii* haplotypes, indicating that the haplotype is of *An. coluzzii* origin and thus that introgession of the allele was from *An. coluzzii* to *An. gambiae*. Haplotype clustering was performed using 500 SNPs around *Cyp9k1*. The Cyp9k1_Dup10 CNV allele was phased by identifying SNPs perfectly correlated with its presence / absence level. Full workings to reproduce this analysis can be found at `https://github.com/vigg-lstm/GAARD_work/blob/main/CNV_analysis/Cyp9k1_Dup10_haplotypes/Cyp9k1_CNV_haplotypes.ipynb`.

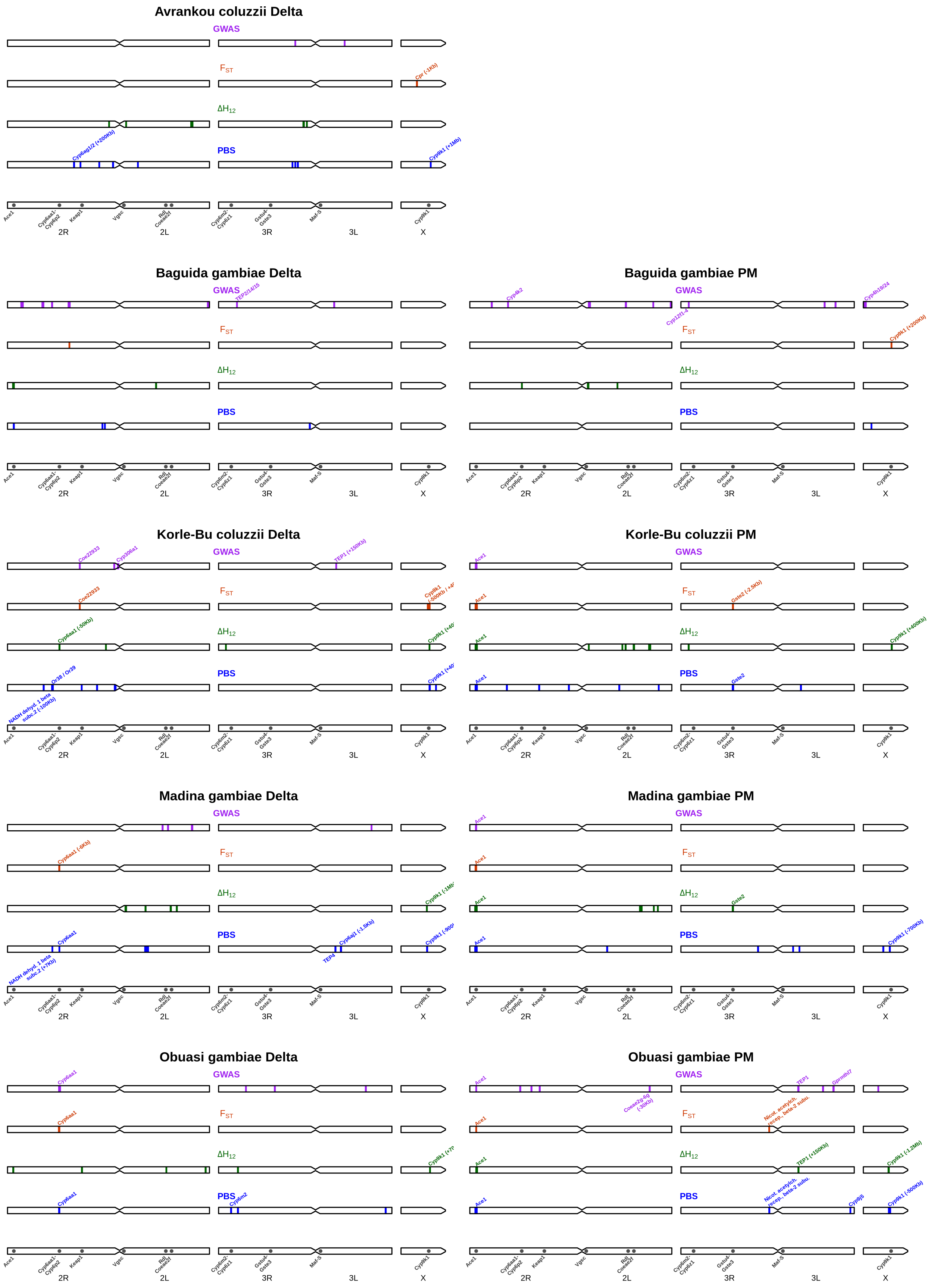

Fig. S4: Genomic regions implicated in insecticide resistance by each of our four approaches. For the global GWAS method, these are 100,000 bp windows which contained at least 10 of the top 1000 significant SNPs. For F_{ST}, these are significant peaks which contained at least one haplotype significantly positively associated with resistance (Supplementary Data S2). For ΔH_{12} and PBS, these are significant positive peaks (ie: indicating stronger signals of selection in resistant compared to susceptible samples). Regions are annotated with genes discussed in the manuscript as possibly causing the signal. Genomic distances in brackets indicate the distance of the peak either to the left (-) or right (+) of the gene in question.

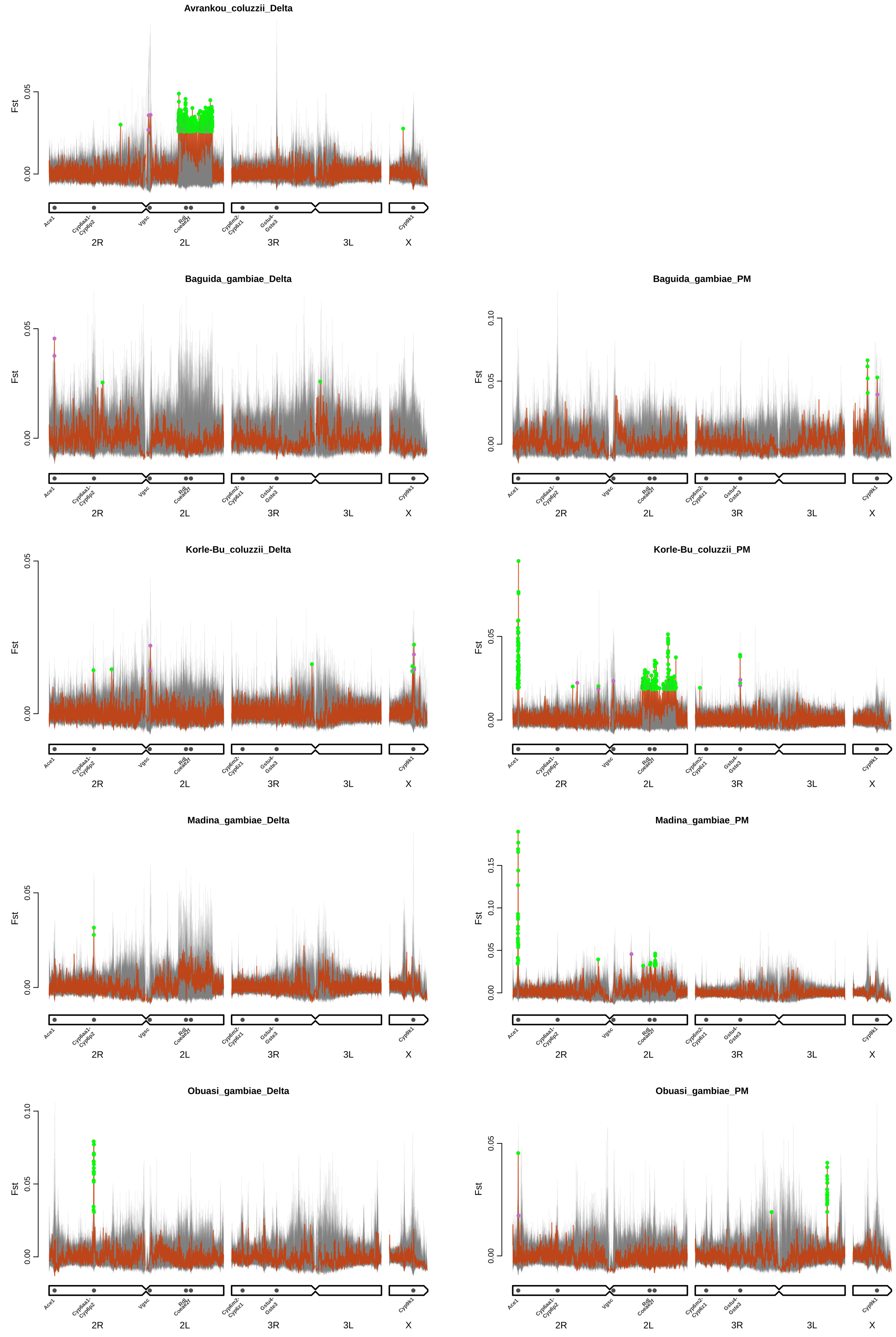

Fig. S5: F_{ST} between resistant and susceptible individuals in each sample set, calculated in 1000 SNP windows. Red line indicates F_{ST}, grey lines in background show results from 200 randomisations in which phenotype labels were permuted. Regions of extended haplotype homozygosity can cause spurious peaks in F_{ST}, which are captured by the randomisations (eg., peaks around Ace1 in Deltamethrin sample sets). Windows identified as peaks were considered "significant" (green points) if their F_{ST} value fell above the 99th centile of the randomisations for that window (ie: $P < 0.01$) and non-significant (purple points) otherwise.

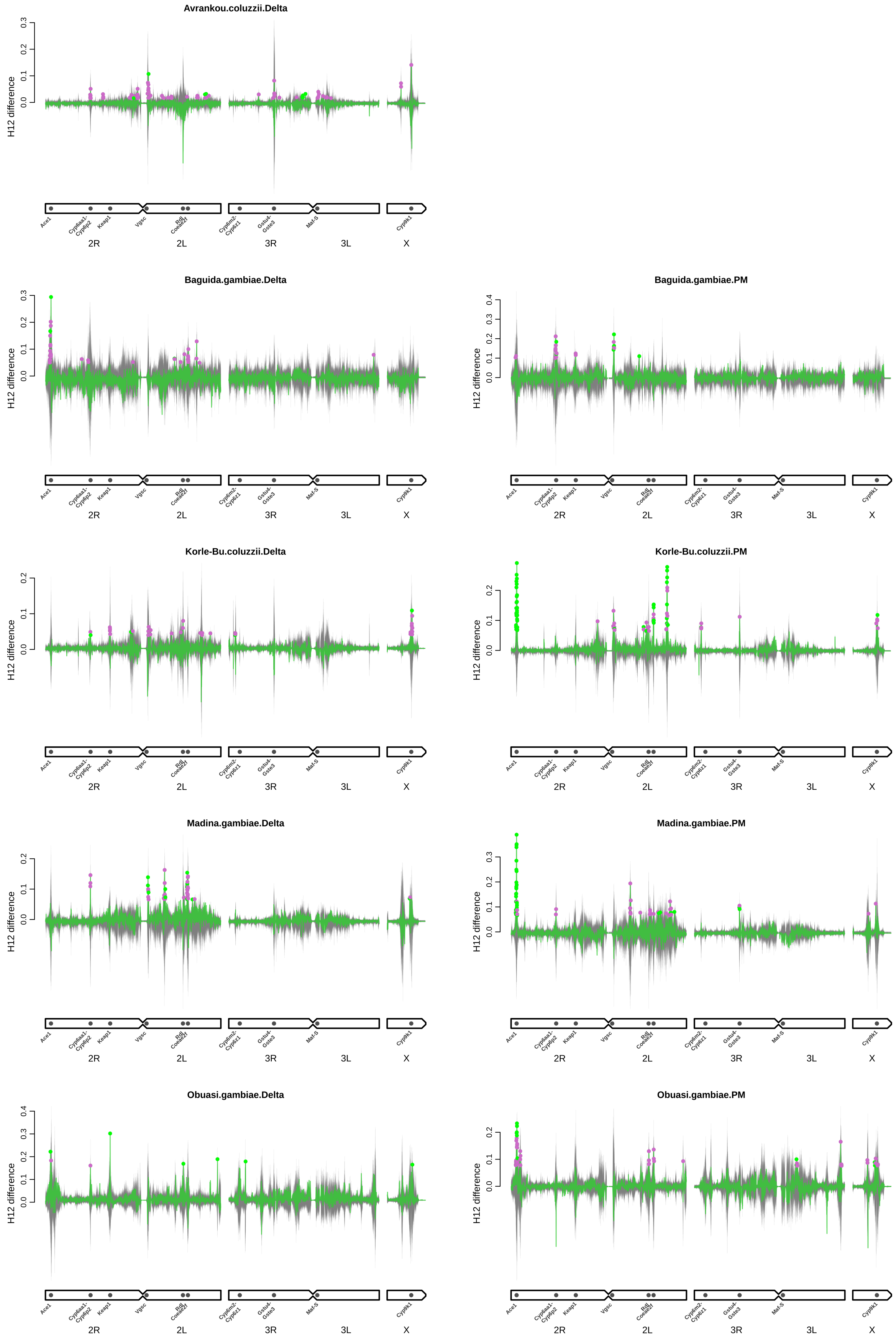

Fig. S6: Difference in H_{12} between resistant and susceptible sub-samples (ΔH_{12}) in each sample set, calculated in 1000 SNP windows. Green line indicates ΔH_{12}, grey lines in background show results from 200 randomisations in which phenotype labels were permuted. Regions of extended haplotype homozygosity can cause spurious peaks in ΔH_{12}, which are captured by the randomisations. Windows identified as positive peaks were considered "significant" (green points) if their ΔH_{12} value fell above the 99th centile of the randomisations for that window (ie: $P < 0.01$) and non-significant (purple points) otherwise.

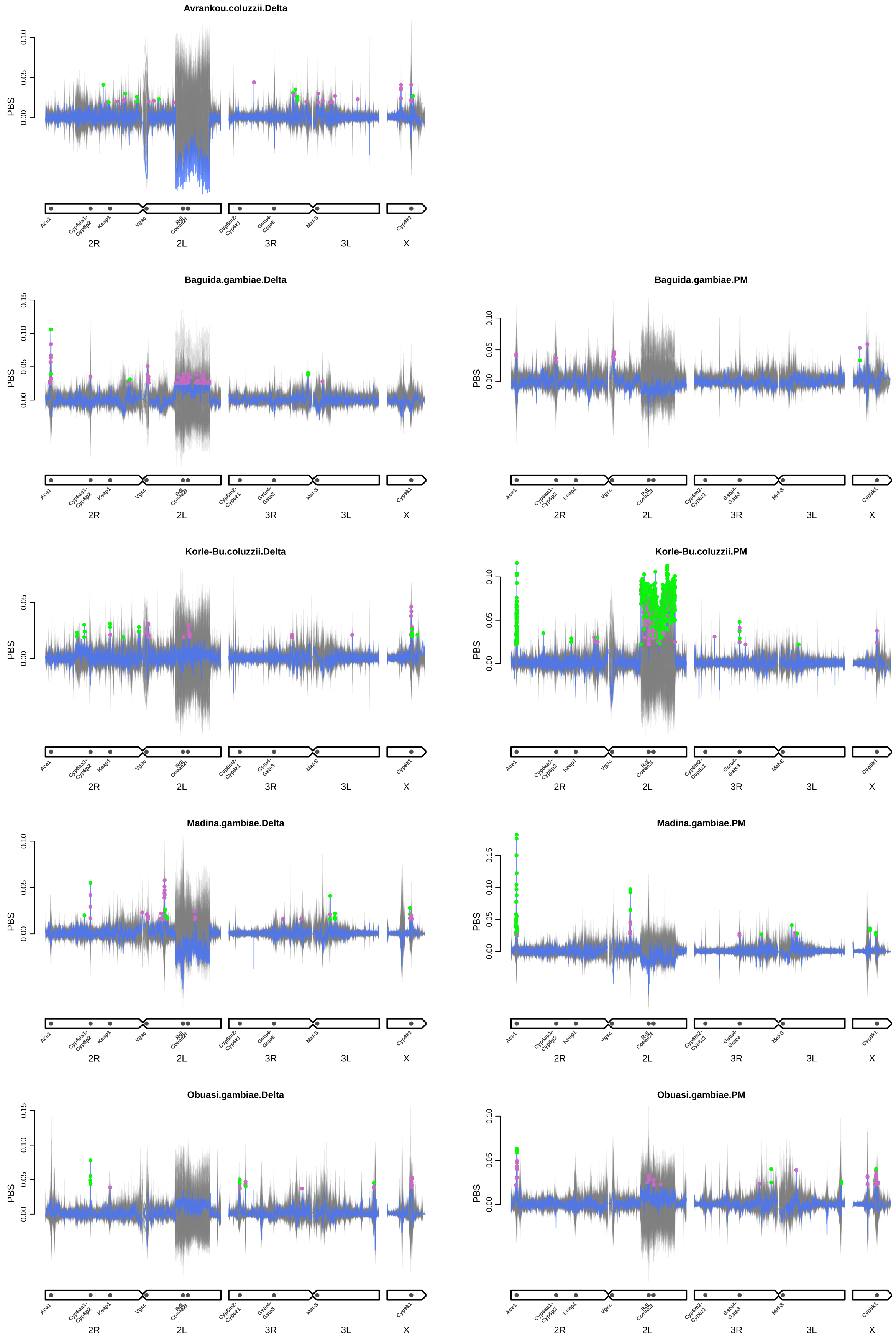

Fig. S7: PBS between resistant and susceptible individuals in each sample set, calculated in 1000 SNP windows. Orange line indicates PBS, grey lines in background show results from 200 randomisations in which phenotype labels were permuted. Regions of extended haplotype homozygosity can cause spurious peaks in PBS which are captured by the randomisations. Windows identified as positive peaks were considered "significant" (green points) if their PBS value fell above the 99th centile of the randomisations for that window (ie: P $<$ 0.01) and non-significant (purple points) otherwise.

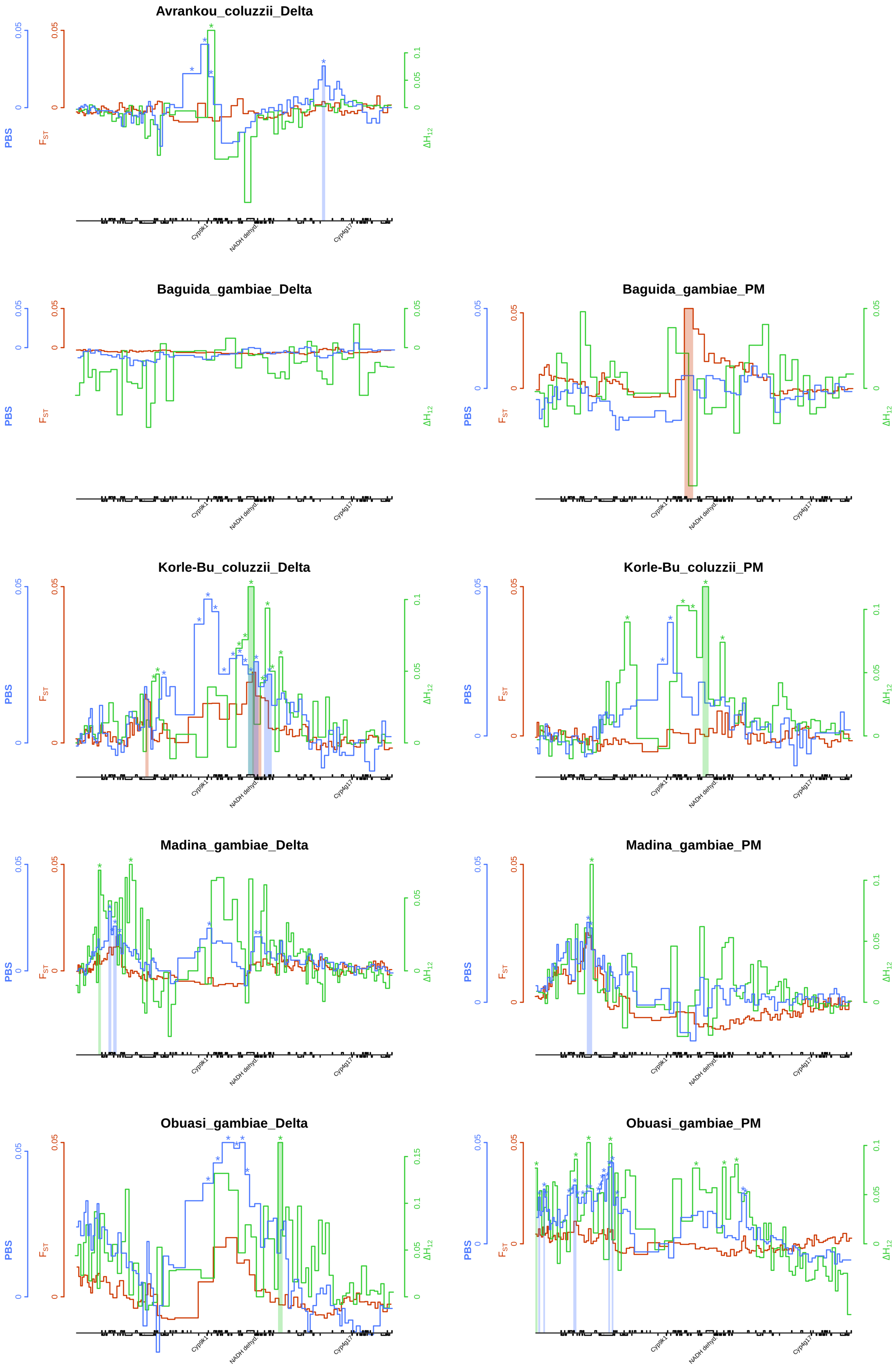

Fig. S8: Caption on next page

Fig. S8 caption: Genomic windows of phenotypic association around *Cyp9k1* were never at the *Cyp9k1* locus itself. Plot shows F_{ST} (red), ΔH_{12} (green) and PBS (blue), with shaded rectangles extending to the bead plot below indicating peaks that were significantly associated with resistance phenotype based on phenotype randomisations (there are the peaks summarised genome-wide in figure S4). Asterisks denote ΔH_{12} and PBS peaks determined by outlier analysis, with many being non-significant according to phenotype randomisations. These peaks may be caused by the presence of a selective sweep in the region, regardless of whether the sweep is associated with the phenotype. The positions of *Cyp9k1*, NADH dehydrogenase (ubquinone) 1 β subcomponent 1 ("NADH dehyd.") and *Cyp4g17* (discussed in the main text) are shown on the bead plot.

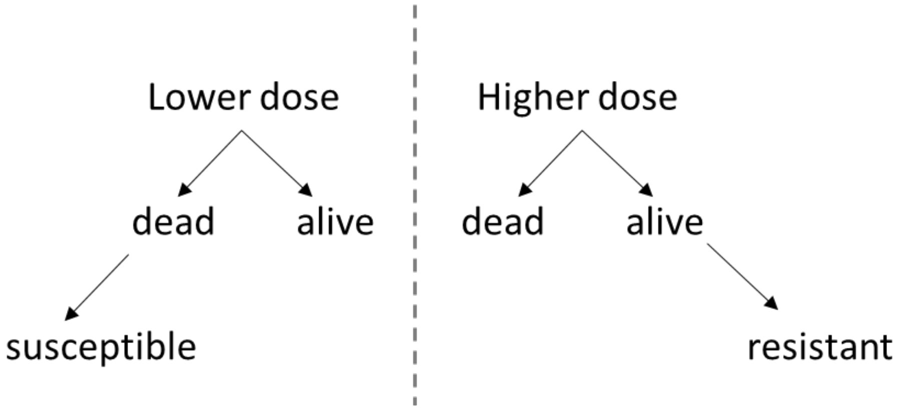

Fig. S9: Schematic diagram illustrating the bioassay strategy to obtain well-separated susceptible and resistant phenotypic groups for whole genome sequencing. Our initial target was to use a lower dose resulting in 20% mortality, and a higher dose resulting in 80% mortality with N=100 samples preserved from each. Though this level of separation was seldom feasible, good separation was generally achieved (see Fig. **S10**).

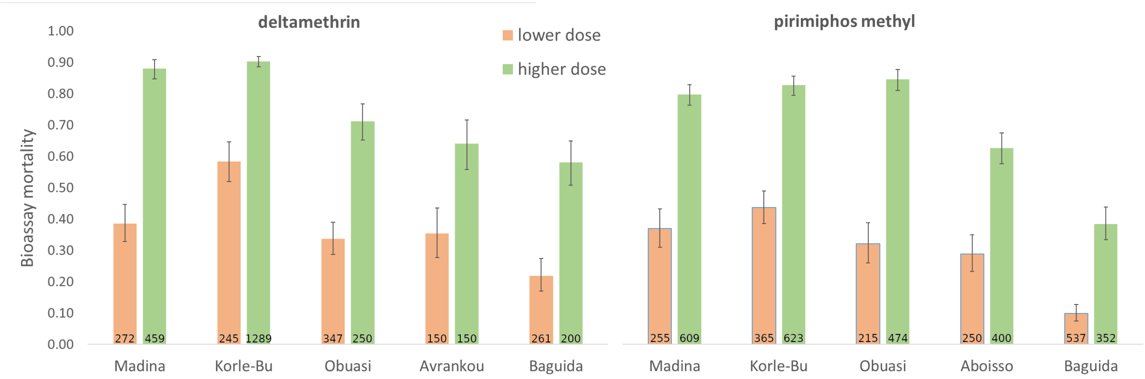

Fig. S10: Mean bioassay mortalities (with 95% binomial confidence intervals) of *An. gambiae* s.l. for the lower and higher doses obtained for each insecticide in the different West African sites sampled. Doses used for each site are shown in Table **S1**, with details of the sibling species composition within each phenotype group in each site. Number of biologically independent samples shown on each bar.

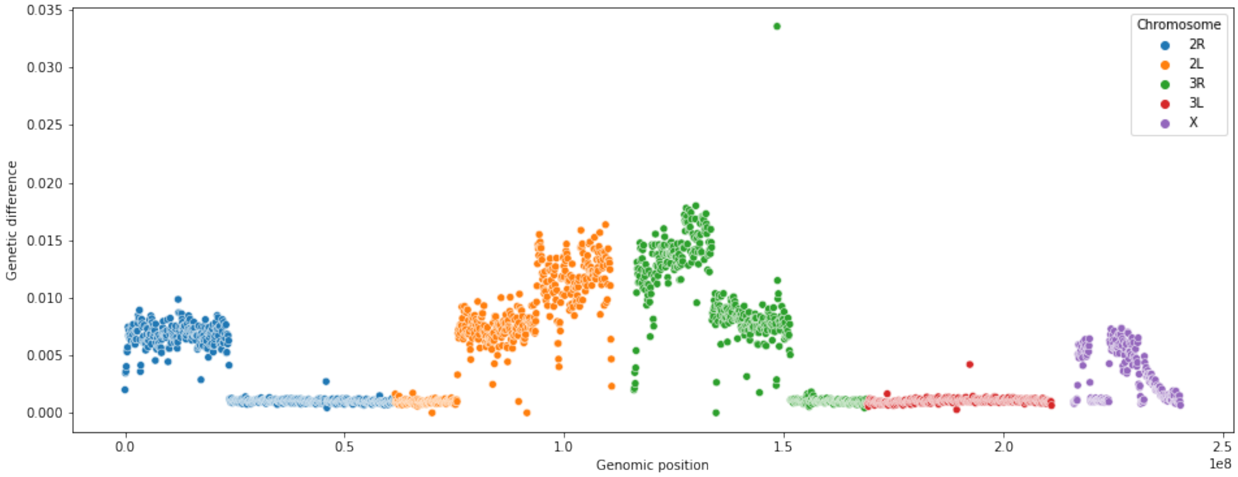

Fig. S11: Genetic difference (calculated as the mean proportion of nucleotide differences across all sites) between a single pair of siblings (WA-0997 and WA-1288, both *An. coluzzii* from Korle-Bu in Ghana) across the genome. Aside from the centromeres, the values of genetic difference occupy one of three states: 0 (genetically identical, where the two individuals inherited the same allele from both their mother and their father, for example the start of Chromosome 2L), around 0.007 (intermediate: where the two individuals inherited the same allele from one of their parents, but not the other, for example the middle of chromosome 2L) and around 0.014 (low: where the two individuals inherited different alleles from both their mother and their father, for example the end of chromosome 2L). These states are invariant over large stretches of the genome (eg: the whole of chromosome 3L is identical between these two individuals) due to the recombination rate per site per generation (usually assumed to be 10^{-8} for modelling purposes) relative to the size of a chromosome (on the order of $5 \cdot 10^{-7}$). Thus, a single chromosome can give an inaccurate picture of relatedness (using chromosome 3L for this pair would make them appear to be clones).

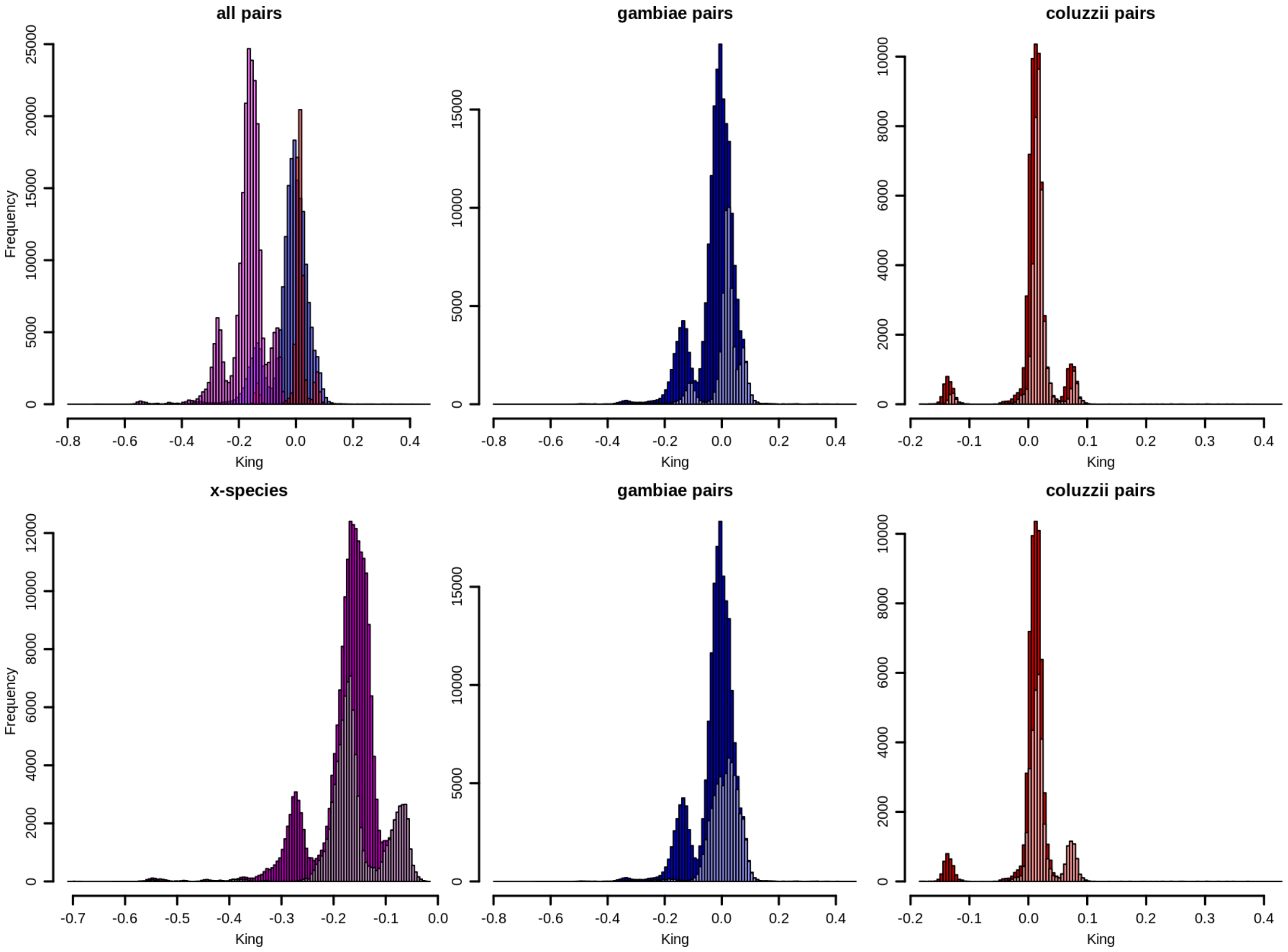

Fig. S12: Histograms of kinship values (KING scores, expected score between full sibs = 0.25, expected score between unrelated individuals = 0) across all sample pairs. **a** Left: Coloured by species (blue = gambiae, red = coluzzii, magenta = cross-species pair). Middle and right: stacked and coloured by whether the pair is of individuals from the same (light colours) or different (dark colours) sampling locations. **b** Stacked and coloured by whether the pair is of individuals with the same (light) or different (dark) 2La karyotype. The three modes of the distribution correspond to the 2La karyotype of the pair, with the right hand mode consisting of pairs of 2La heterozygotes (shared heterozygote genotype produces the largest relatedness values), the main central mode consisting of pairs with the same homozygous karyotype (in dark) or where one individual is heterozygous and the other homozygous (in light), and the left hand mode consisting of pairs with opposite homozygous karyotypes.

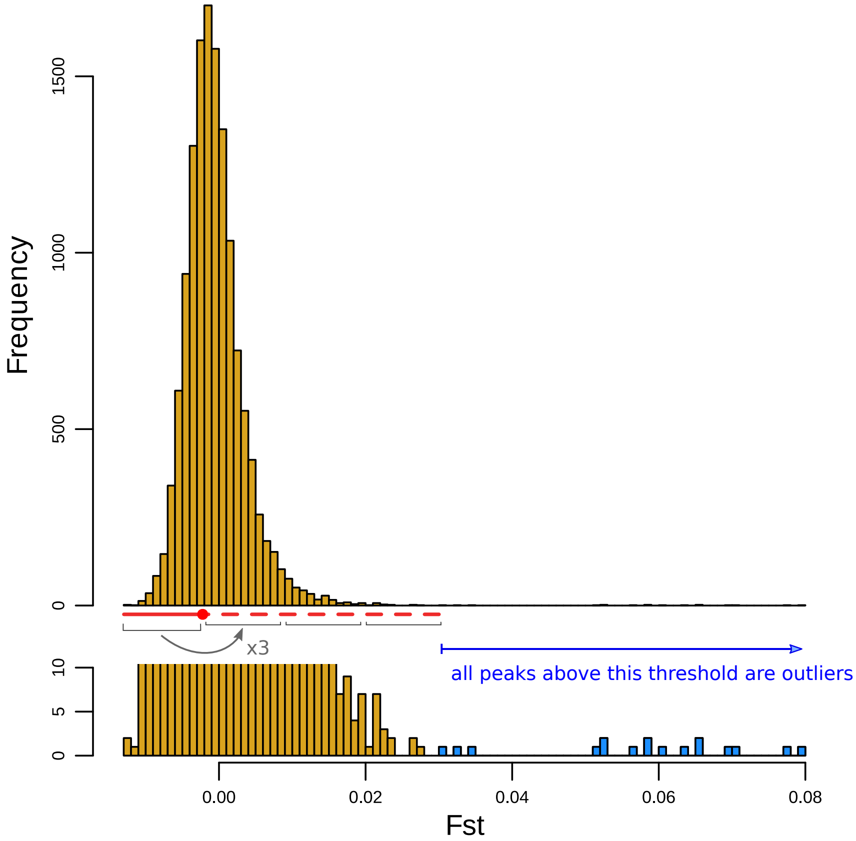

Fig. S13: Historgram of values between phenotype groups in *An. gambiae* from Obuasi exposed to deltamethrin (top) and zoomed in on at the origin of the Y axis to show small outlier peaks (bottom). We first identified the mode of the distribution (red point) and then calculated its distance from the minimum of the distribution (solid red line). We then conservatively took three times this distance to the right of the mode (dashed red line) as our threshold. Any windows with values larger than this (in blue) were considered outliers and thus provisional windows of interest.

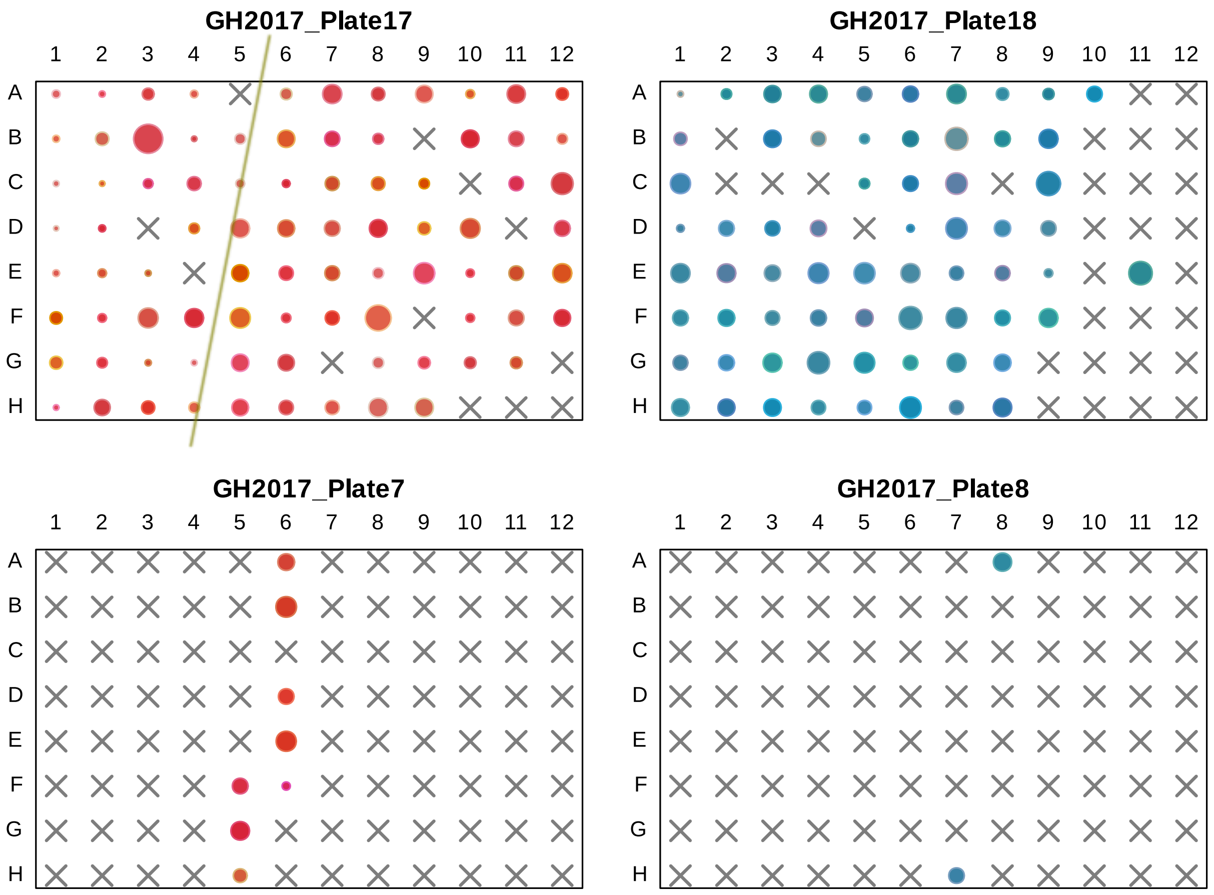

Fig. S14: Layout of levels of *Asaia* reads across DNA storage plates for deltamethrin-exposed samples from Korle-Bu. Size of circle indicates normalised number of Asaia reads. Colour indicates phenotype of sample (blue = alive, red = dead). Crosses indicate wells not used for sequencing. Diagonal line on top left plate separates wells with broadly less *Asaia* signal on the left and more signal on the right.

Table **S1**: Bioassays to segregate females defined as susceptible (dead at lower dose) from resistant (alive at higher dose). All bioassays were performed on *An. gambiae* s.l. with subsequent molecular identification of species within the tested samples performed on a subset of samples (last four columns). Numbers highlighted in bold show the groups chosen for sequencing. Final numbers for each sample set, after QC filtering of sequencing data, are shown in Table 1 (main text). Delta = deltmethrin; PM = pirimiphos-methyl.

			Lower dose details		Higher dose details		*An. gambiae s.l.*		*An. gambiae*		*An. coluzzii*	
**Country**	**Site**	**Insecticide**	**Conc.**	**Time (min)**	**Conc.**	**Time (min)**	**Tested N lower dose**	**Tested N higher dose**	**Dead N**	**Alive N**	**Dead N**	**Alive N**
Benin	Avrankou	Delta	0.5X	60	2X	60	150	150	1	1	**49**	**57**
Ghana	Korle-Bu	Delta	0.5X	60	5X	60	245	1289	0	0	**43**	**58**
	Korle-Bu	PM	0.5X	60	1X	60	365	623	0	0	**68**	**46**
	Madina	Delta	1X	60	10X	60	272	459	**76**	**45**	65	2
	Madina	PM	0.5X	60	1X	60	255	609	**63**	**43**	28	9
	Obuasi	Delta	2X	60	5X	60	347	250	**111**	**68**	1	0
	Obuasi	PM	0.5X	15, 30	0.5X	45, 60	215	474	**69**	**71**	0	0
						75, 90					0	0
Ivory Coast	Aboisso	PM	0.5X	60	1X	60	250	400	**13**	**75**	51	8
Togo	Baguida	Delta	2X	60	10X	60	261	200	**56**	**81**	1	1
	Baguida	PM	0.5X	45	0.5X	90	537	352	**44**	**58**	6	1
