# Supplementary material for: Genome-wide association studies reveal novel loci associated with pyrethroid and organophosphate resistance in Anopheles gambiae and Anopheles coluzzii
Source: Nat Commun. 2023 Aug 16;14:4946. doi: 10.1038/s41467-023-40693-0 (PMC10432508; doi:10.1038/s41467-023-40693-0)
Supplement: Supplementary file 3 — Reporting Summary [file 41467_2023_40693_MOESM3_ESM.pdf]

nature portfolio

Corresponding author(s): Eric Lucas

Last updated by author(s): May 10, 2023

Reporting Summary

Nature Portfolio wishes to improve the reproducibility of the work that we publish. This form provides structure for consistency and transparency in reporting. For further information on Nature Portfolio policies, see our Editorial Policies and the Editorial Policy Checklist.

Statistics

For all statistical analyses, confirm that the following items are present in the figure legend, table legend, main text, or Methods section.

n/a	Confirmed	
☐	☒	The exact sample size (*n*) for each experimental group/condition, given as a discrete number and unit of measurement
☐	☒	A statement on whether measurements were taken from distinct samples or whether the same sample was measured repeatedly
☐	☒	The statistical test(s) used AND whether they are one- or two-sided<br>*Only common tests should be described solely by name; describe more complex techniques in the Methods section.*
☐	☒	A description of all covariates tested
☐	☒	A description of any assumptions or corrections, such as tests of normality and adjustment for multiple comparisons
☐	☒	A full description of the statistical parameters including central tendency (e.g. means) or other basic estimates (e.g. regression coefficient) AND variation (e.g. standard deviation) or associated estimates of uncertainty (e.g. confidence intervals)
☐	☒	For null hypothesis testing, the test statistic (e.g. *F*, *t*, *r*) with confidence intervals, effect sizes, degrees of freedom and *P* value noted<br>*Give P values as exact values whenever suitable.*
☒	☐	For Bayesian analysis, information on the choice of priors and Markov chain Monte Carlo settings
☐	☒	For hierarchical and complex designs, identification of the appropriate level for tests and full reporting of outcomes
☒	☐	Estimates of effect sizes (e.g. Cohen's *d*, Pearson's *r*), indicating how they were calculated

Our web collection on statistics for biologists contains articles on many of the points above.

Software and code

Policy information about availability of computer code

Data collection	No code was used for data collection
Data analysis	Code and pipelines used for the processing of raw sequencing data can be found at github.com/malariagen/vector-ops and malariagen.github.io/vetor-data-ag/methods.html .<br>Code for the downstream analysis in this project can be found on github: github.com/vigg-lstm/GAARD_work<br>Software and packages used are R v4.1.2, fdrtoold v1.2.17, Python v3.8.12, Bracken v2.5

For manuscripts utilizing custom algorithms or software that are central to the research but not yet described in published literature, software must be made available to editors and reviewers. We strongly encourage code deposition in a community repository (e.g. GitHub). See the Nature Portfolio guidelines for submitting code & software for further information.

Data

Policy information about availability of data

All manuscripts must include a data availability statement. This statement should provide the following information, where applicable:

- Accession codes, unique identifiers, or web links for publicly available datasets
- A description of any restrictions on data availability
- For clinical datasets or third party data, please ensure that the statement adheres to our policy

The sequencing data generated in this study have been deposited in the ENA short read archive database, with all accession codes provided in Supplementary Data

S1. The accession number for the genome assembly to which the reads were aligned is GCA_000005575.1. The bracken analysis was performed using the GTDB database release 89: https://data.ace.uq.edu.au/public/gtdb/data/releases/release89. The bioassay data generated in this study are provided in Supplementary Data S1.

Research involving human participants, their data, or biological material

Policy information about studies with human participants or human data. See also policy information about sex, gender (identity/presentation), and sexual orientation and race, ethnicity and racism.

Reporting on sex and gender	This study did not involve human participants
Reporting on race, ethnicity, or other socially relevant groupings	This study did not involve human participants
Population characteristics	This study did not involve human participants
Recruitment	This study did not involve human participants
Ethics oversight	This study did not involve human participants

Note that full information on the approval of the study protocol must also be provided in the manuscript.

Field-specific reporting

Please select the one below that is the best fit for your research. If you are not sure, read the appropriate sections before making your selection.

☐ Life sciences ☐ Behavioural & social sciences ☒ Ecological, evolutionary & environmental sciences

For a reference copy of the document with all sections, see nature.com/documents/nr-reporting-summary-flat.pdf

Ecological, evolutionary & environmental sciences study design

All studies must disclose on these points even when the disclosure is negative.

Study description	Genome-wide association study of insecticide resistance in Anopheles gambiae. Phenotypes were resistant (alive after insecticide exposure) and susceptible (dead after exposure). Samples were obtained from six locations and 969 individual mosquitoes were sequenced.
Research sample	The research samples was a group of female Anopheles gambiae mosquitoes, collected as larvae from the field in West Africa (Benin, Cote d'Ivoire, Ghana, Togo) and reared in the lab until the adult age of 3-5 days. Samples were chosen to determine insecticide resistance using WHO tube assays, which require 3-5 day-old females. Locations were chosen based to the known presence of resistance in these mosquito populations. Sequencing data were aligned to the Anopheles gambiae PEST genome assembly, AgamP4 (accession GCA_000005575.1). Bracken analysis was conducted with the GTDB database: https://data.ace.uq.edu.au/public/gtdb/data/releases/release89.
Sampling strategy	Samples were collected as larvae and raised to adulthood in the laboratory, as is standard procedure for resistance testing. Sample sizes were based on the sequencing capacity allocated by the Anopheles gambiae 1000 genomes project. The obtained sample sizes were able to detect known signals of resistance, indicating that sample size was sufficient for association testing.
Data collection	Resistance bioassays were recorded with pen and paper, and conducted by Constant Edi and Benjamin Koudou (Côte d'Ivoire); Luc S. Djogbénou and Adandé Medjigbodo (Benin); Guillaume Ketoh (Togo); Alexander Egyir-Yawson , John Essandoh, Sam Dadzie and Joseph Chabi (Ghana). Whole genome sequencing was performed by the Anopheles gambiae 1000 genomes consortium and data recorded on the servers at the Wellcome Trust Sanger Institute.
Timing and spatial scale	Mosquito collection dates:<br>Aboisso:<br>Avrankou: 29/09/17 - 10/11/17<br>Baguida: 07/12/2017 - 31/01/2018<br>Korle-Bu: 02/02/18 - 15/02/18<br>Madina: 02/11/17 - 26/12/17<br>Obuasi: 04/09/17 - 26/10/17<br>Spatial scale: All samples were collected within a 5km radius.
Data exclusions	Data were excluded if sequencing failed or if samples were found to be males after sequencing. The failure of some samples at the sequencing stage was anticipated, but the presence of males was not. For the GWAS, we also excluded all but one sample from each sib group, in order to remove the confounding effects of relatedness. This was also not anticipated.
Reproducibility	Conducting a single replicate of this study was a huge undertaking and thus not reproduced.

Randomization	Samples were divided into phenotypic groups (resistant / susceptible) based on the outcome of the phenotypic assay. So other grouping was necessary for the experiment.
Blinding	Blinding was not relevant to this study as all mosquitoes were subjected to the same phenotypic assay, and the genotyping was done by whole-genome sequencing and a pre-established analytical pipeline which was not affected by the phenotypic group that each mosquito belonged to.

Did the study involve field work? ☒ Yes ☐ No

Field work, collection and transport

Field conditions	Field work was conducted at breeding sites of Anopheles gambiae. Temperature and rainfall were not recorded.
Location	Latitude, longitude of the collection sites were:<br>Madina: 5.683, -0.166<br>Obuasi 6.2, -1.683<br>Baguida 6.161, 1.314<br>Aboisso 5.467, -3.2<br>Korle-Bu 5.537, -0.24<br>Avrankou 6.55, 2.667<br>Elevation and water depth were not recorded.
Access & import/export	Samples were collected by local researchers in established collection sites, using a highly targeted method of dipping handheld nets into pools to collect mosquito larvae, thus avoiding damage to the environment and other species. These researchers also exported the samples and are included in the list of authors. Samples were imported into the UK under import license IMP/GEN/2014/06 issued by the Department for Environment, Food and Rural Affairs (24th March 2014).
Disturbance	No disturbance was caused by the study

Reporting for specific materials, systems and methods

We require information from authors about some types of materials, experimental systems and methods used in many studies. Here, indicate whether each material, system or method listed is relevant to your study. If you are not sure if a list item applies to your research, read the appropriate section before selecting a response.

Materials & experimental systems

n/a	Involved in the study	
☒	☐	Antibodies
☒	☐	Eukaryotic cell lines
☒	☐	Palaeontology and archaeology
☐	☒	Animals and other organisms
☒	☐	Clinical data
☒	☐	Dual use research of concern
☒	☐	Plants

Methods

n/a	Involved in the study	
☒	☐	ChIP-seq
☒	☐	Flow cytometry
☒	☐	MRI-based neuroimaging

Animals and other research organisms

Policy information about studies involving animals; ARRIVE guidelines recommended for reporting animal research, and Sex and Gender in Research

Laboratory animals	No laboratory animals were used in this study.
Wild animals	Anopheles gambiae and Anopheles coluzzii larvae of unknown age were collected in the field by dipping and transported back to the lab in water containers. Males were kept in cages until natural death. Females were used for bioassays and collected when they were 3-5 days old. Females that were not killed by exposure to the insecticide used in the bioassays were killed by placing them in the freezer. Mosquitoes are pest species and therefore should be killed rather than released.
Reporting on sex	Mosquito sex was initially determined morphologically and only females were used for bioassays because insecticide resistance is a phenotype that is only of interest in female mosquitoes (they are the ones that can transmit malaria). After sequencing, sex was confirmed molecularly by comparing the sequencing coverage between the sex chromosome and autosomes. This identified four males that had been incorrectly assigned morphologically. Overall, the study involved 1254 females and 4 males. We report data for females only, as they are the sex of interest for this study as explained above.

Field-collected samples	Field-collected larvae were kept in trays of clean water and transferred to 30x30 cm plastic-frame mesh cages at adulthood. Larvae were fed on tetramin fish food and adults provided with 10% sugar-water ad libitum. Rearing conditions were ambient temperature and a day-night cycle of 12h-12h. At the end of the study, all mosquitoes that remained alive were killed by placing in the freezer.
Ethics oversight	The only animals used in this study were mosquitoes, which are not subject to ethical restrictions

Note that full information on the approval of the study protocol must also be provided in the manuscript.
