# Supplementary material for: Genome-wide association studies reveal novel loci associated with pyrethroid and organophosphate resistance in Anopheles gambiae and Anopheles coluzzii
Source: Nat Commun. 2023 Aug 16;14:4946. doi: 10.1038/s41467-023-40693-0 (PMC10432508; doi:10.1038/s41467-023-40693-0)
Supplement: Supplementary file 4 — Description of Additional Supplementary Files [file 41467_2023_40693_MOESM4_ESM.pdf]

Description of Additional Supplementary Files

Supplementary Data 1
Description: Sample metadata and accession numbers.

Supplementary Data 2
Description: details of the SNPs that distinguish the haplotype clusters identified in the Fst windows of interest.

Supplementary Data 3
Description: Genomic extent of CNV alleles presented in Supplementary Figure 2.
